# Knockdown of *NEAT1* restrained the malignant progression of glioma stem cells by activating microRNA *let-7e*

**DOI:** 10.18632/oncotarget.11403

**Published:** 2016-08-19

**Authors:** Wei Gong, Jian Zheng, Xiaobai Liu, Jun Ma, Yunhui Liu, Yixue Xue

**Affiliations:** ^1^ Department of Neurobiology, College of Basic Medicine, China Medical University, Shenyang 110122, People's Republic of China; ^2^ Institute of Pathology and Pathophysiology, China Medical University, Shenyang 110122, People's Republic of China; ^3^ Department of Neurosurgery, Shengjing Hospital of China Medical University, Shenyang 110004, People's Republic of China; ^4^ Liaoning Research Center for Translational Medicine in Nervous System Disease, Shenyang 110004, People's Republic of China

**Keywords:** lncRNAs, *NEAT1*, glioma stem cells, *let-7e*, *NRAS*

## Abstract

Nuclear paraspeckle assembly transcript 1 (*NEAT1*), a long non-coding RNA, promotes oncogenesis in various tumors, including human gliomas. Herein, we studied the expression and function of *NEAT1* in glioma stem cells (GSCs). Quantitative real-time PCR demonstrated that *NEAT1* was upregulated in GSCs. *NEAT1* knockdown inhibited GSC cell proliferation, migration and invasion and promoted GSC apoptosis. A potential binding region between *NEAT1* and microRNA *let-7e* was confirmed by dual-luciferase assays. Upregulation of *NEAT1* reduced the expression of *let-7e*, and there was reciprocal repression between *NEAT1* and *let-7e* in an Argonaute 2-dependent manner. *Let-7e* expression was lower expression in glioblastoma tissues and GSCs than in normal brain tissues and cells. Restoration of *let-7e* suppressed tumor function by inhibiting proliferation, migration and invasion while promoting apoptosis in GSCs. *NEAT1* knockdown and *let-7e* overexpression both reduced NRAS protein expression. *NRAS* was identified as a direct target of *let-7e* and promoted oncogenesis in GSCs. As *NEAT1* promoted oncogenesis by downregulating *let-7e* expression, both of these genes could be considered for application in glioma therapy.

## INTRODUCTION

Glioma is one of the most prevalent and aggressive primary brain tumors in adults. Despite treatment with advanced therapeutic strategies, patients with this disease only have a median survival of 15 months [1]. Glioma stem cells (GSCs) are a subpopulation of glioma cells, and are characterized by self-renewal, promotion of angiogenesis, and multi-differentiation [2]. GSCs are important contributors to the malignant progression of glioma, from its development to therapeutic resistance and recurrence [3]. Therefore, it is urgent to discover the molecular mechanisms by which GSCs are maintained, as this would provide a new focus for the development of glioma treatments.

Long non-coding RNAs (lncRNAs, ~200nt) are a class of RNAs that do not encode proteins [4]. LncRNAs are widely expressed in various human somatic tissues, and are involved in diverse cellular events, including epigenetic regulation, gene transcription, mRNA processing and gene translation [5]. LncRNAs are ubiquitously dysregulated in tumor cells and have crucial regulatory roles in the malignant progression of tumor cells, such as promotes or suppresses proliferation, migration and invasion, and apoptosis [6]. Nuclear paraspeckle assembly transcript 1 (*NEAT1*) is a 4-kb lncRNA, and has been reported to localize to the nucleus, where it serves as a core component of the paraspeckle sub-organelles [7–9]. *NEAT1* is upregulated and has important functions in a variety of cancers including glioma, such as favors cell proliferation, migration and invasion and impaired apoptosis [10–13]. However, whether *NEAT1* is associated with the malignant progression of GSCs remains unclear.

MicroRNAs (miRNAs, ~22nt) are a group of small non-coding RNAs with aberrant expression in various tumors. MiRNAs are involved in diverse biological processes, such as cell growth, migration, apoptosis and differentiation, by binding to the 3′-UTRs of mRNAs [14, 15]. The *let-7* family of miRNAs comprises 13 members, one of which is *let-7e* [16]. The *let-7* family is characterized by regulating diverse biological processes in cancer cells, including inhibited proliferation and promoted apoptosis [17]. The downregulation of *let-7e* is known to be a prognostic marker of squamous-cell lung carcinoma [18]. Further, *let-7e* is repressed in medulloblastoma [19]. *Let-7e* expression is reduced in deep vein thrombosis and prevents cell endothelial progenitor function by binding to FASLG [20]. Remarkably, *let-7e* can bind to MMP9 and induce adipose-derived stem cell differentiation [21]. However, the expression and function of *let-7e* in GSCs remain unclear.

The *RAS* gene is recognized to encode three isoforms - HRAS, KRAS and NRAS [22] - and is involved in diverse cellular events and signaling pathways. *NRAS* is approximately 4.3 kb in length and is aberrantly expressed in many tumors, including colorectal cancer and cutaneous melanoma [23–25]. However, little has been studied about the function of NRAS in GSCs.

In the current study, we sought to determine the expression and function of *NEAT1*, *let-7e* and *NRAS* in glioma tissues and GSCs. We also investigated the interactions among them in the regulation of GSC malignant behavior and the potential molecular pathways involved.

## RESULTS

### Isolation and identification of GSCs

**Figure 1:**
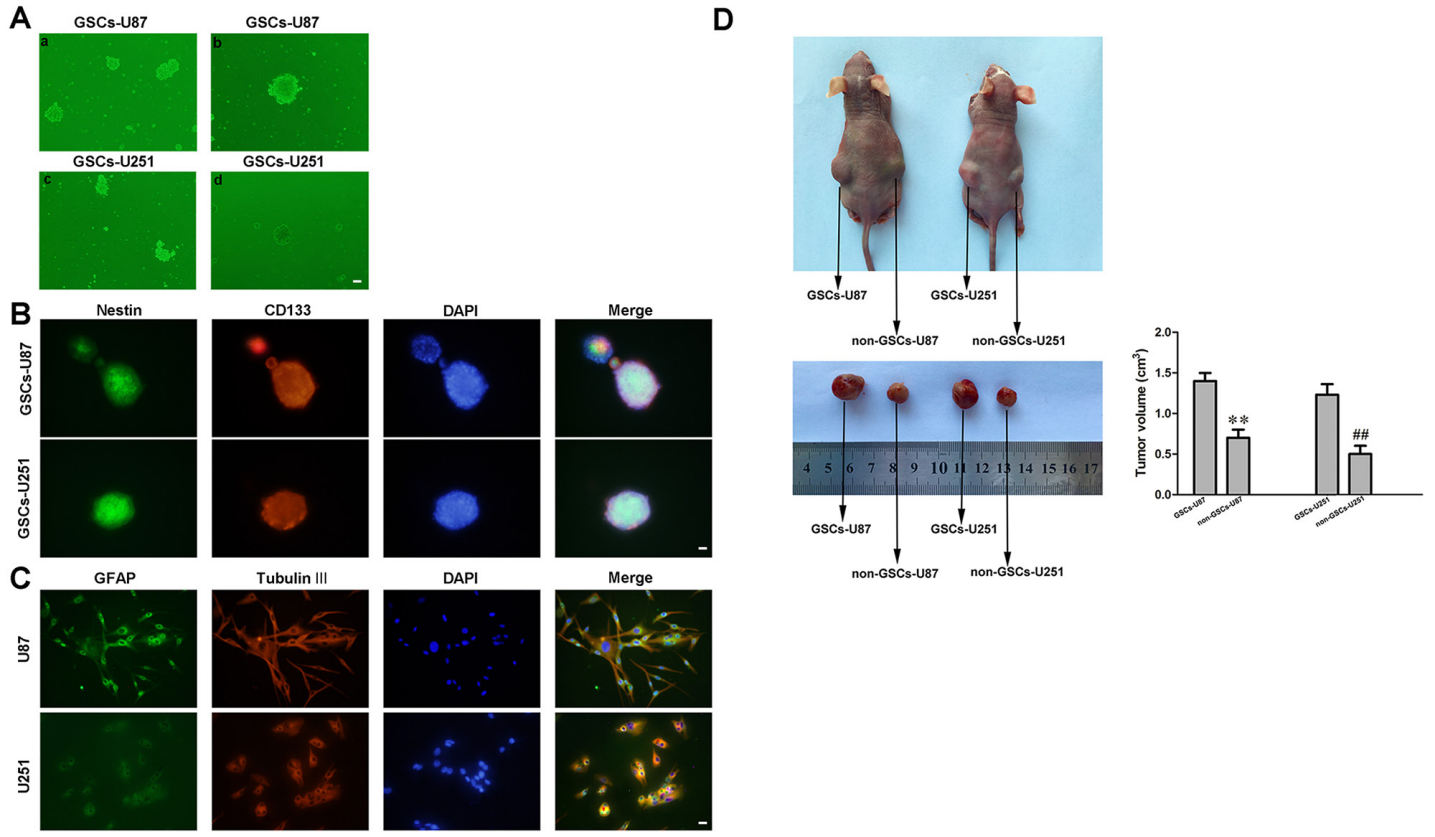
Isolation and identification of GSCs **A. a** and **c**: U87 and U251 glioma cells formed spheres in serum-free medium. **b** and **d**: single cells formed spheres again in a second-round sphere-forming assay. **B.** GSCs-U87 and GSCs-U251 stained for Nestin (green) and CD133 (red) by immunofluorescence analysis. **C.** GSC-U87 and GSC-U251 spheres were differentiated and then stained for GFAP (green) and beta-tubulin III (red) by immunofluorescence. **D.** Subcutaneously implanted GSCs-U87 or GSCs-U251 formed xenografts in nude mice. Data are presented as the mean ± SD (n=5, each group). ^**^P<0.01 vs. GSC-U87 group; ^##^*P*<0.01 vs. GSC-U251 group.

Cells isolated from U87 and U251 cell lines were cultured in serum-free medium and allowed to form cell spheres (Figure 1A (a, c)). In an effort to verify the self-renewing abilities of the cells, we harvested the spheres and performed a second round of the sphere-forming assay. As expected, spheres were generated again from single cells (Figure 1A (b, d)). The positive staining of Nestin and CD133 confirmed that most cells within the spheres expressed these neural stem cell lineage markers on their membranes (Figure 1B). Moreover, the cell spheres stained positively for glial fibrillary acidic protein (GFAP) and beta-tubulin-III (lineage markers), suggesting that they were undergoing typical morphological differentiation towards astrocytic and neuronal lineages (Figure 1C). Further, GSCs-U87 and GSCs-U251 induced larger xenografted tumors in mice than non-GSCs, indicating their stronger tumorigenicity (Figure 1D).

### *NEAT1* was upregulated while *let-7e* was downregulated in GSCs

**Figure 2:**
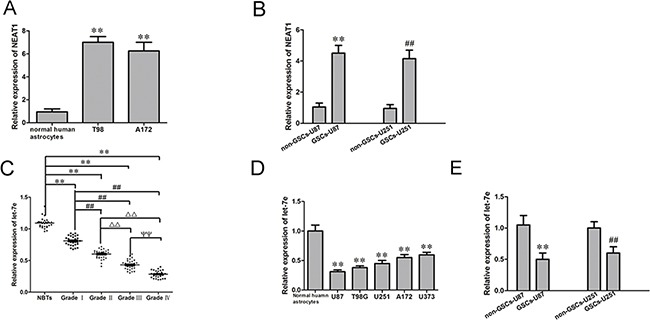
*NEAT1* and *let-7e* levels in glioma tissues and GSCs **A.** Expression of *NEAT1* in human normal astrocytes and glioma cell lines (n=5, each group). **P<0.01 vs. Normal human astrocyte group. **B.** Expression of *NEAT1* in non-GSCs and GSCs (n=5, each group). ^**^P<0.01 vs. non-GSC-U87 group; ^##^*P*<0.01 vs. non-GSC-U251 group. **C.** Expression of *let-7e* in glioma tissues of different grades and NBTs (n=30, each group). ^**^P<0.01 vs. NBT group; ^##^*P*<0.01 vs. Grade I group; ^ΔΔ^*P*<0.01 vs. Grade II group;^ΨΨ^*P*<0.01 vs. Grade III group. **D.** Expression of *let-7e* in human normal astrocytes and glioma cell lines (n=5, each group). ^**^P<0.01 vs. normal human astrocyte group. **E.** Expression of *let-7e* in non-GSCs and GSCs (n=5, each group). ^**^P<0.01 vs. non-GSC-U87 group; ^##^*P*<0.01 vs. non-GSC-U251 group. All data are presented as the mean ± SD

As previously reported, *NEAT1* was upregulated in glioblastoma (GBM) tissues [10]. Also, we found *NEAT1* was upregulated in GBM U87 and U251 cell lines (Figure 2A and 2B). Quantitative real-time PCR (qRT-PCR) was conducted to determine the expression of *NEAT1* in two additional glioma cell lines and GSCs. As shown in Figure 2A and 2B, *NEAT1* expression was significantly upregulated in T98, A172, GSC-U87 and GSC-U251 cells. On the contrary, *let-7e* expression was significantly lower in GBM tissues and GBM cell lines than in normal brain tissues and normal human astrocytes, and correlated negatively with the glioma pathological grade (Figure 2C–2E). These results suggested that *NEAT1* promotes oncogenesis in GSCs, while *let-7e* functions as a tumor suppressor.

### Knockdown of *NEAT1* impaired the malignant progression of GSCs

**Figure 3:**
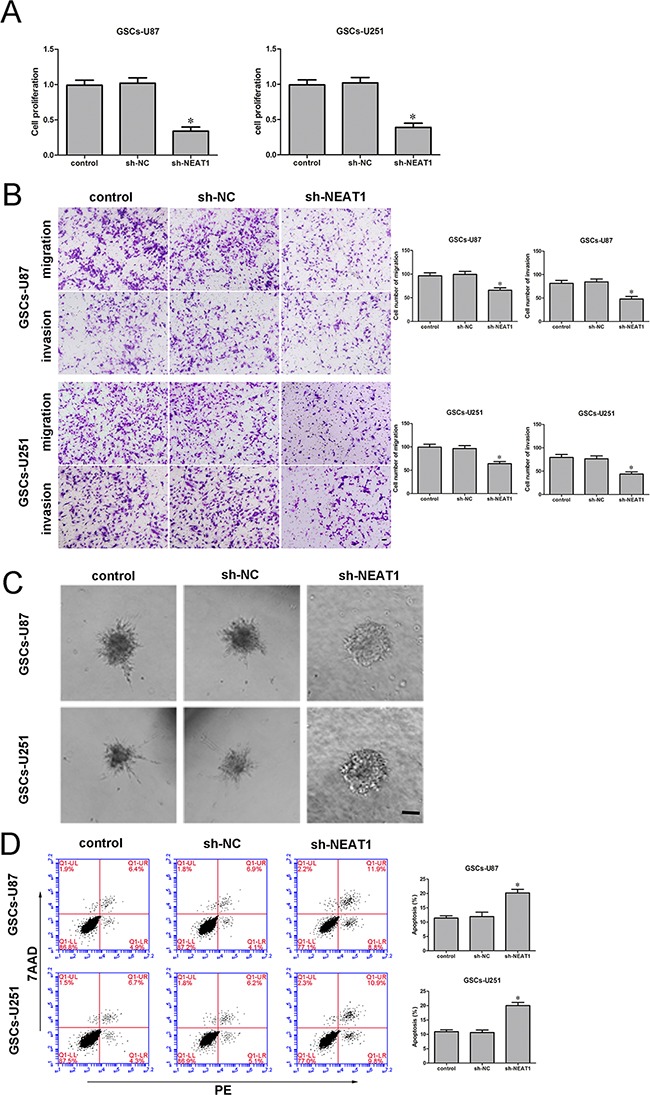
Knockdown of *NEAT1* restrained cell proliferation, migration and invasion and promoted apoptosis of GSCs **A.** ACCK-8 assay was used to determine the effect of *NEAT1* on GSC proliferation. **B.** Quantification of the migration and invasion of *NEAT1*-knockdown GSCs. Representative images and accompanying statistical plots are presented. **C.** 3D Spheroid-based tumor migration assays of the effect of *NEAT1* expression on GSC migration. Scale bars, 60 μm. **D.** Flow cytometry analysis of the effect of *NEAT1* knockdown on GSCs. Data are presented as the mean ± SD (n=5, each group). ^*^P<0.05 vs. sh-NC group. Scale bars, 20 μm.

To determine the effect of *NEAT1* on GSCs, we divided cells into three groups: the control group, sh-NC group (transfected with the sh-NC plasmid), sh-*NEAT1* group (transfected with the sh-*NEAT1* plasmid) and the sh-NC group (transfected with the sh-NC plasmid). The Cell Counting Kit-8 (CCK-8) assay indicated that GSC proliferation was lower in the *NEAT1*-knockdown group than in the sh-NC group (Figure 3A). The migration and invasion of GSCs were also significantly lower in the sh-*NEAT1* group than in the sh-NC group (Figure 3B). Moreover, GSCs treated with sh-*NEAT1* exhibited weaker migration abilities than control cells in 3D Spheroid-based tumor migration assays (Figure 3C). Flow cytometry analysis revealed that the inhibition of *NEAT1* significantly increased GSC apoptosis (Figure 3D). These results indicated that *NEAT1* might act as an oncogene in GSCs.

### *Let-7e* functioned as a tumor suppressor

**Figure 4:**
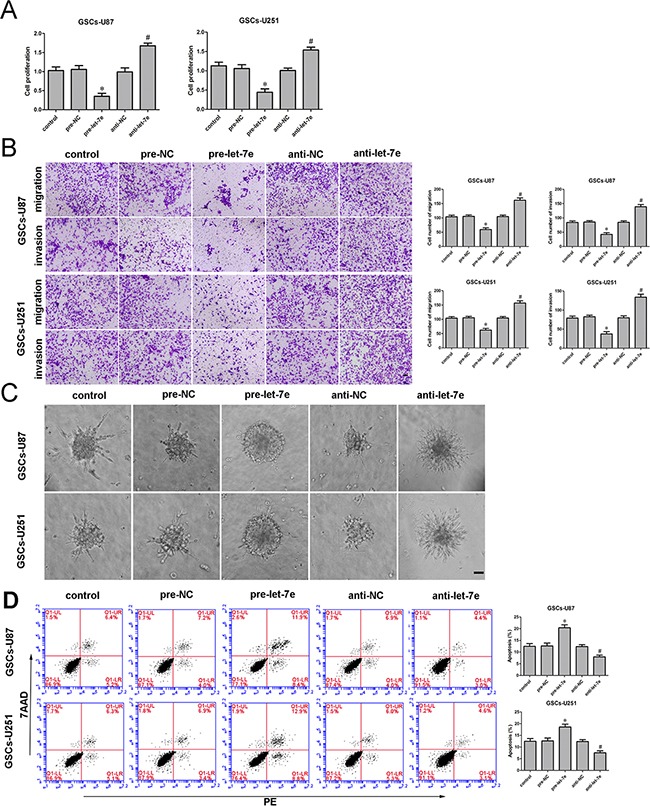
Restoration of *let-7e* inhibited cell proliferation, migration and invasion and facilitated apoptosis of GSCs **A.** The CCK-8 assay was employed to determine the effect of *let-7e* on GSC proliferation. **B.** Quantification of GSC migration and invasion in groups according to *let-7e* expression. Representative images and accompanying statistical plots are presented. **C.** 3D Spheroid-based tumor migration assays of the effect *let-7e* expression on GSC migration. Scale bars, 60 μm. **D.** Flow cytometry analysis of GSCs in groups according to *let-7e* expression. Data are presented as the mean ± SD (n=5, each group). ^*^P<0.05 vs. pre-NC group; *^#^*P<**0.05 vs. anti-NC group. Scale bars, 20 μm.

Similarly, to determine the effect of *let-7e* on GSCs, we divided cells into five groups: the control group, pre-NC group (transfected with *let-7e* agomir NC), pre-*let-7e* group (transfected with the *let-7e* agomir), anti-NC group (transfected with *let-7e* antagomir NC) and anti-*let-7e* (transfected with the *let-7e* antagomir). A CCK-8 assay revealed that GSC proliferation was lower in the *let-7e* overexpression group (pre-*let-7e*) than in the pre-NC group (Figure 4A). Transwell assays were conducted to assess the effects of *let-7e* overexpression on the invasiveness and migratory abilities of GSCs. The migration and invasion of GSCs were lower in the pre-*let-7e* group than in the pre-NC group (Figure 4B). Similarly, GSC spheroid migration was attenuated (Figure 4C) and apoptosis was enhanced in the *let-7e* overexpression group relative to the anti-*let-7e* group. Thus, it is conceivable that *let-7e*, in contrast to *NEAT1*, functions as a tumor suppressor in GSCs (Figure 4D).

### *NEAT1* is a direct target of *let-7e*

**Figure 5:**
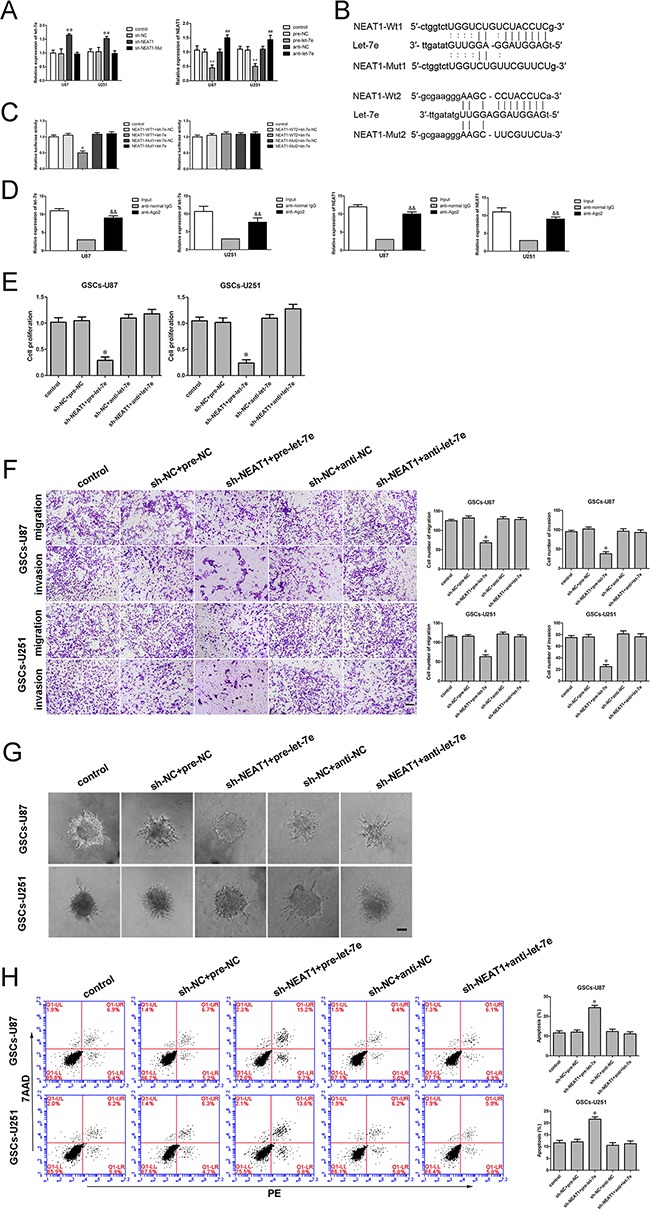
Binding and reciprocal repression between *let-7e* and *NEAT1* determined GSC malignant behavior **A.** qRT-PCR analysis demonstrating the negative correlation between *let-7e* and *NEAT1* expression in GSCs. ^**^P<0.01 vs. sh-NC group; ^**^P<0.01 vs. pre-NC group; ^##^*P*<0.01 vs. sh-NC group. **B.** *NEAT1* harbored two putative *let-7e* binding sites; the designed mutant sequences are indicated. **C.** Dual-luciferase reporter assay of HEK 293T cells co-transfected with *NEAT1*-Wt1 (or *NEAT1*-Wt2) and *let-7e*-NC; *NEAT1*-Wt1 (or *NEAT1*-Wt2) and *let-7e*; *NEAT1*-Mut1 (or *NEAT1*-Mut2) and *let-7e*-NC; or *NEAT1*-Mut1 (or *NEAT1*-Mut2) and *let-7e*. ^*^P<0.05 vs. *NEAT1*-Wt1+*let-7e*-NC group. **D.** *Let-7e* was identified in the *NEAT1*-RISC complex. *NEAT1* and *let-7e* levels were measured by qRT-PCR. ^&&^*P* < 0.01 vs. the anti-normal IgG group. **E.** The CCK-8 assay was applied to evaluate the effects of *NEAT1* and *let-7e* on GSC proliferation. **F.** Quantification of the migration and invasion of GSCs in groups according to *NEAT1* and *let-7e* expression. Representative images and accompanying statistical plots are presented. **G.** 3D Spheroid-based tumor migration assays of the effect of *NEAT1* and *let-7e* co-transfection on GSC migration. Scale bars, 60 μm. **H.** Flow cytometry analysis of GSCs in groups according to *NEAT1* and *let-7e* expression. ^*^P<0.05 vs. sh-NC+pre-NC group. Scale bars, 20 μm. For A, C, D, E and H, data are presented as the mean ± SD (n=5, each group).

There is increasing evidence that lncRNAs could be competing endogenous RNAs (ceRNAs) or molecular sponges in down-regulating the expression and biological functions of miRNAs, Using a bioinformatics database (Starbase), we determined that *NEAT1* harbors two putative binding sites for *let-7e* (Figure 5B). To validate our hypothesis that *let-7e* could directly bind to *NEAT1*, we first measured the expression of *let-7e* in sh-*NEAT1* GSCs by qRT-PCR. *Let-7e* expression was elevated in the sh-*NEAT1* group but not in the sh-*NEAT1*-Mut group. In contrast, *NEAT1* expression was reduced in the pre-*let-7e* group (Figure 5A). Further, dual-luciferase gene reporter assays were used to assess the binding sites of *NEAT1* and *let-7e*. The luciferase activity was significantly lower in the *NEAT1*-Wt1+pre-*let-7e* group than in the control group (Figure 5C), while the luciferase activity did not differ between the *NEAT1*-Mut1+pre-*let-7e* group and the control group, suggesting that binding site 1 between *NEAT1* and *let-7e* was functional.

An RNA-binding protein immunoprecipitation (RIP) assay was conducted to determine whether *NEAT1* and *let-7e* were in the expected RNA-induced silencing complex (RISC). We explored RNA levels by qRT-PCR, and found *NEAT1* and *let-7e* immunoprecipitated with Argonaute 2 were repressed than those in the control group, respectively (Figure 5D). Thus, *NEAT1* inhibition was confirmed to restore *let-7e* expression in a RISC-dependent manner, and there was a reciprocal repression feedback loop between *NEAT1* and *let-7e*.

### *NEAT1* inhibition hindered the malignant progression of GSCs by upregulating *let-7e*

Further, to explore the mechanism whereby *NEAT1* promoted malignant behavior in GSCs by attenuating *let-7e* expression, we divided cells into five groups: the control group, the sh-NC+pre-NC group (cells stably expressing sh-NC, co-transfected with pre-NC), sh-*NEAT1*+pre-*let-7e* group (cells stably expressing sh-*NEAT1*, co-transfected with pre-*let-7e*), sh-NC+anti-NC group (cells stably expressing sh-NC, co-transfected with anti-NC) and sh-*NEAT1*+anti-*let-7e* group (cells stably expressing sh-*NEAT1*, co-transfected with anti-*let-7e*). GSC proliferation was markedly lower in the sh-*NEAT1*+pre-*let-7e* group than in the sh-NC+pre-NC group (Figure 5E). Moreover, the numbers of migrating and invading GSCs were significantly lower in the sh-*NEAT1*+pre-*let-7e* group than in the control group (Figure 5F). In addition, GSCs treated with sh-*NEAT1*+pre-*let-7e* exhibited weaker migration abilities than controls in 3D Spheroid-based tumor migration assays (Figure 5G). The apoptotic ratio of GSCs with knockdown of *NEAT1* and overexpression of *let-7e* was robustly elevated compared with that in the sh-NC+pre-NC group (Figure 5H). These results indicated that knockdown of *NEAT1* hindered malignant behavior in GSCs by upregulating *let-7e*.

### NRAS was upregulated in glioma tissues and GSCs, and facilitated GSC proliferation, migration and invasion and reduced GSC apoptosis

**Figure 6:**
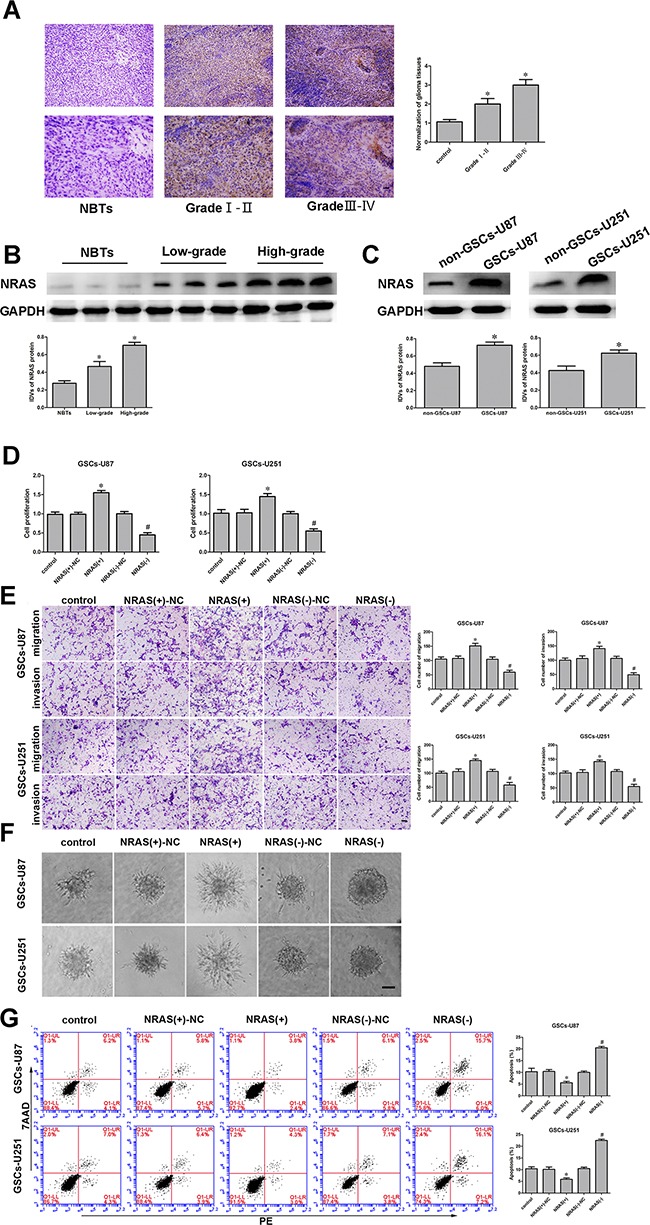
*NRAS* was upregulated in glioma tissues, glioma cell lines and GSCs And *NRAS* promoted cell proliferation, migration and invasion and inhibited apoptosis of GSCs. **A.** Immunohistochemistry of NRAS protein levels in non-tumorous brain, Grade I-II glioma, and Grade III-IV glioma tissues. Original magnification: 100× (above), 200× (below). Scale bar, 50 μm. **B.** NRAS protein expression in non-tumorous brain tissues and glioma tissues, with GAPDH as an endogenous control. Representative protein expression and integrated light density values of NRAS in non-tumorous brain tissues, low-grade glioma tissues (WHO I-II), and high-grade glioma tissues (WHO III-IV) are shown. Data are presented as the mean ± SD (n=15, each group). ^*^P<0.05 vs. non-tumorous brain tissue group. **C.** Western blot of NRAS expression in non-GSCs and GSCs, with GAPDH as an endogenous control. ^*^P<0.05 vs. non-GSC group. **D.** The CCK-8 assay was employed to determine the effect of NRAS on GSC proliferation. **E.** Quantification of GSC migration and invasion upon *NRAS* inhibition. Representative images and accompanying statistical plots are presented. **F.** 3D Spheroid-based tumor migration assays of the effect of *NRAS* expression on GSC migration. Scale bars, 60 μm. **G.** Flow cytometry analysis of the effects of *NRAS* overexpression or downregulation on GSCs. Data are presented as the mean ± SD (n=5, each group). ^*^P<0.05 vs. *NRAS* (+)-NC group; *^#^*P<**0.05 vs. *NRAS* (−)-NC group. Scale bars, 20 μm.

*NRAS* was previously identified as an oncogene in several cancers. NRAS protein levels in normal brain tissues, glioma tissues and GSCs were investigated by immunohistochemistry and Western blot. As shown in Figure 6A, NRAS localized to the cytoplasm and was upregulated in glioma tissues compared with normal brain tissues. Similarly, the Western blot revealed greater protein levels of NRAS in glioma tissues and GSCs than in normal brain tissues and non-GSCs (Figure 6B and 6C). Thus, we inferred that NRAS promotes the malignant progression of GSCs. NRAS has been reported to act as an oncogene in various tumor cells. To determine the effect of NRAS on GSCs, we divided cells into five groups: the control group, *NRAS* (+)-NC group (transfected with the empty plasmid), *NRAS* (+) group (transfected with the *NRAS* full-length plasmid), *NRAS* (−)-NC group (transfected with the empty plasmid) and *NRAS* (−) group, (transfected with the short-hairpin *NRAS* plasmid). CCK-8 assays revealed that GSC proliferation was enhanced in the *NRAS* overexpression group compared to the *NRAS* (+)-NC group (Figure 6D). Transwell assays demonstrated that GSC migration and invasion were greater in the *NRAS* overexpression group than in the *NRAS* (+)-NC group (Figure 6E). Further, overexpression of *NRAS* induced the migration of GSCs in a 3D Spheroid-based tumor migration assay (Figure 6F). *NRAS* overexpression also significantly reduced GSC apoptosis (Figure 6G).

### NRAS was involved in the *NEAT1*/*let-7e*-dependent malignant progression of GSCs

**Figure 7:**
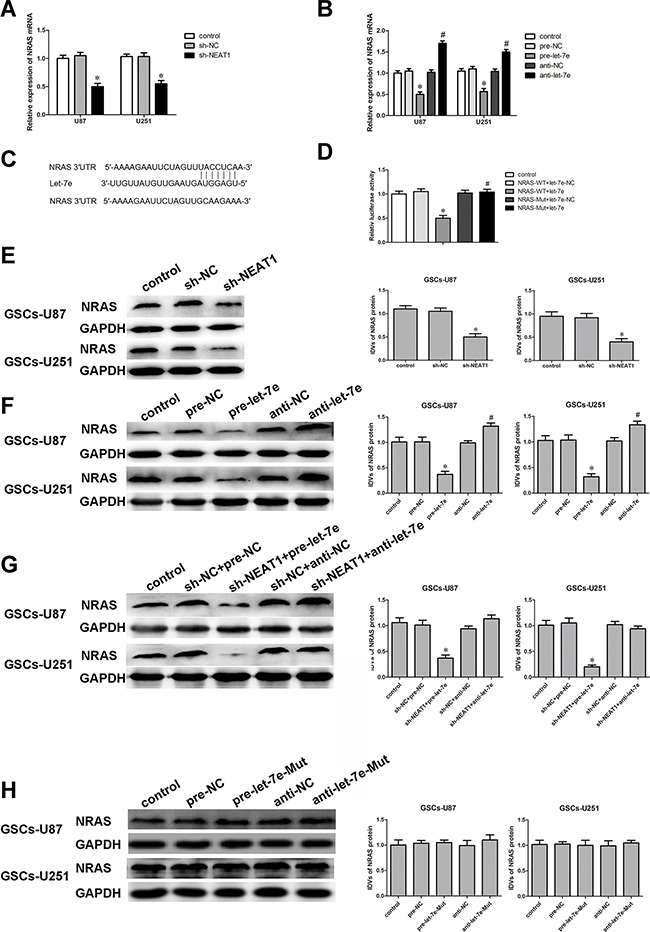
*NRAS* was a target gene of *Let-7e* And the expression of NRAS was regulated by both *NEAT1* and *let-7e*. **A.** qRT-PCR analysis of the effect of *NEAT1* on *NRAS* mRNA expression in GSCs. ^*^P<0.05 vs. sh-NC group. **B.** qRT-PCR analysis of the effect of *let-7e* on *NRAS* mRNA expression in GSCs. ^*^P<0.05 vs. pre-NC group; ^#^*P*<0.05 vs. anti-NC group. **C.** *NRAS* harbored one putative *let-7e* binding site, the designed mutant sequence is indicated. **D.** Dual-luciferase reporter assay of HEK 293T cells co-transfected with *NRAS*-Wt and *let-7e*-NC; *NRAS*-Wt and *let-7e*; *NRAS*-Mut and *let-7e*-NC; or *NRAS*-Mut and *let-7e*. ^*^P<0.05 vs. *NRAS*-Wt+*let-7e*-NC group. **E.** Western blot analysis for NRAS in *NEAT1*-knockdown GSCs, with GAPDH as an endogenous control. ^*^P<0.05 vs. sh-NC group. **F.** Western blot analysis for NRAS in *let-7e*-overexpression and *let-7e*-inhibition GSCs, with GAPDH as an endogenous control. ^*^P<0.05 vs. pre-NC group; ^#^*P*<0.05 vs. anti-NC group. **G.** Western blot analysis for NRAS in GSCs co-transfected with sh-*NEAT1* and pre-*let-7e* or anti-*let-7e*, with GAPDH as an endogenous control. ^*^P<0.05 vs. sh-NC+pre-NC group. **H.** Western blot analysis for NRAS in *let-7e*-Mut-overexpression or *let-7e*-Mut-inhibition GSCs, with GAPDH as an endogenous control. For G-K, data are presented as the mean ± SD (n=5, each group).

Bioinformatics databases (Targetscan, Starbase and miRanda) predicted that several genes would be downstream targets of *let-7e*, including *NRAS*. We first determined the effect of *let-7e* on the protein levels of NRAS by Western blot, and found that NRAS expression was significantly downregulated, among several downstream molecules of *let-7e* predicted by the bioinformatics databases. Next, the mRNA and protein levels of *NRAS* were detected in GSCs treated with sh-*NEAT1*, pre-*let-7e* or anti-*let-7e* by qRT-PCR and Western blot. *NRAS* mRNA and protein levels were lower in the sh-*NEAT1* group than in the sh-NC group (Figure 7A and 7E). On the contrary, GSCs treated with anti-*let-7e* exhibited higher mRNA and protein levels of *NRAS* than those treated with anti-NC (Figure 7B and 7F). NRAS expression was significantly lower in the sh-*NEAT1*+pre-*let-7e* group than in the sh-NC+pre-NC group (Figure 7G).

Having confirmed that both *NEAT1* and *let-7e* influence the malignant behavior of GSCs, we performed a dual-luciferase reporter assay to verify the putative binding site (Figure 6C) between *let-7e* and *NRAS*. Luciferase activity was lower in the *NRAS*-Wt+*let-7e* group than in the *NRAS*-Wt+*let-7e*-NC group, but did not differ significantly between the *NRAS*-Mut+*let-7e* and *NRAS*-Mut+*let-7e*-NC groups (Figure 7D).

To further confirm the binding sites between *let-7e* and *NRAS*, we mutated the expected binding sequence in *let-7e*. As shown in Figure 7H, NRAS protein levels did not change in the pre-*let-7e*-Mut group than in the pre-NC group.

### *Let-7e* impaired *NRAS*-induced malignant behavior in GSCs by binding to its 3′-UTR

To discover whether *let-7e* prevented GSC malignant evolution by binding to a specific sequence in *NRAS*, we investigated the extent of proliferation, migration, invasion and apoptosis in GSCs stably expressing *let-7e*+*NRAS* (non-3′UTR). We divided cells into four groups: the *let-7e*-NC+*NRAS*-NC group (cells stably expressing pre-NC, co-transfected with the *NRAS*-NC plasmid), *let-7e*+*NRAS*-NC group (cells stably expressing pre-*let-7e*, co-transfected with *NRAS*-NC), *let-7e*+*NRAS* group (cells stably expressing pre-*let-7e*, co-transfected with *NRAS* (+)) and *let-7e*+*NRAS* (non-3′UTR) group (cells stably expressing *let-7e*, co-transfected with *NRAS* (without 3′-UTR) plasmid).

**Figure 8:**
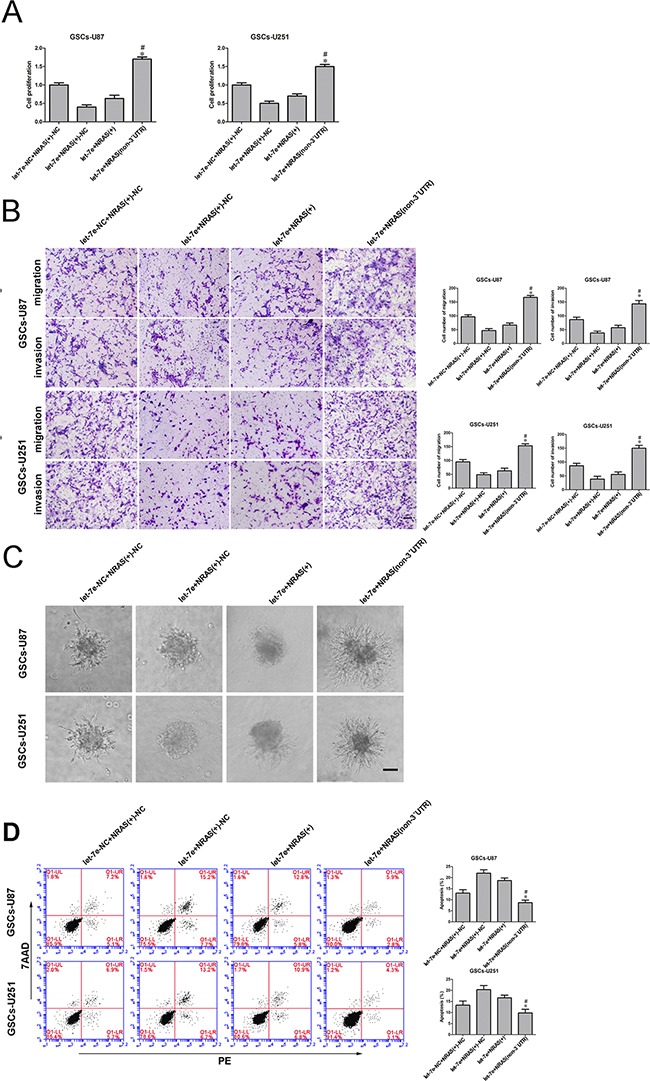
*Let-7e* inhibited GSC malignant progression by binding to the *NRAS* 3′-UTR **A.** The CCK-8 assay was used to evaluate the effects of *let-7e* and *NRAS* (+) on U87 and U251 cell proliferation. **B.** Quantification of cell migration and invasion in groups according to *let-7e* and *NRAS* (+)*-NC* expression. Representative images and accompanying statistical plots are presented. **C**. Migration abilities of GSCs in groups according to *let-7e* and *NRAS* (+) expression. Scale bars, 60 μm. **D.** Flow cytometry analysis of GSCs in groups according to *let-7e* and *NRAS* (+) expression. Data are presented as the mean ± SD (n=5, each group). ^*^P<0.05 vs. *let-7e*+*NRAS* group. Scale bars, 40 μm.

The proliferation of GSCs was significantly greater in the *let-7e*+*NRAS* (non-3′UTR) group than in the *let-7e*+*NRAS* group (Figure 8A). Further, in Transwell assays, there were significantly more migrating and invading GSCs in the *let-7e*+*NRAS* (non-3′UTR) group than in the *let-7e*+*NRAS* group (Figure 8B). Further, GSCs treated with *let-7e*+*NRAS* (non-3′UTR) had stronger migration abilities (Figure 8C) and a lower extent of apoptosis (Figure 8D) than those treated with *let-7e*+*NRAS*.

## DISCUSSION

In this study, we demonstrated that *NEAT1* was upregulated in GSCs. *NEAT1* inhibition impaired the malignant behavior of GSCs and attenuated *NRAS* expression. On the contrary, *let-7e* expression was downregulated in glioma tissues and GSCs. Restoration of *let-7e* suppressed proliferation, migration and invasion, promoted apoptosis, and reduced *NRAS* expression in GSCs. Moreover, *let-7e* was found to bind to *NEAT1* in a sequence-specific manner, and there was reciprocal repression between *let-7e* and *NEAT1,* possibly induced by the RISC. *NRAS* was identified as a direct target of *let-7e* and was involved in the *NEAT1*-induced malignant progression of GSCs. Further, NRAS was confirmed to promote oncogenesis in GSCs by stimulating cell proliferation, migration and invasion while inhibiting apoptosis.

There is much evidence that lncRNAs are aberrantly expressed in various tumors. Due to their involvement in tumorigenesis and cancer progression, lncRNAs could be used as diagnostic or prognostic biomarkers and potential therapeutic targets. *NEAT1* is an lncRNA confirmed to be upregulated in gliomas, and promotes cell proliferation, migration and invasion while suppressing apoptosis in glioma U87 and U251 cells [10].

GSCs are a subgroup of glioma cells characterized by self-renewal, promotion of angiogenesis and multi-differentiation. Conventional therapies against glioma may be limited mainly due to the existence of GSCs, which facilitate the recurrence, drug-resistance, rapid growth, invasion and metastasis of glioma [2]. Therefore, therapy against GSCs has become an urgent and promising field. In the current study, we found that *NEAT1* was upregulated in GSCs, suggesting that it might promote GSC progression. Indeed, inhibition of *NEAT1* reduced the malignant progression of GSCs. Consistent with our results, *NEAT1* was reported to be upregulated in non-small cell lung cancers and to promote lung cancer cell growth [26]. Likewise, the overexpression of *NEAT1* contributes to the malignant behavior of laryngeal squamous cell cancer by downregulating the *miR-107/CDK6* pathway [27]. We further examined the expression of *NRAS*, which promotes oncogenesis in various cancers, and found that *NEAT1* knockdown significantly reduced *NRAS* expression in GSCs. However, whether NRAS is also involved in the *NEAT1*-induced enhancement of GSC progression needs to be further investigated.

We also found that *let-7e*, a conserved miRNA, was downregulated in glioma tissues and GSCs, which suggested that *let-7e* might be associated with GSC progression. As reported earlier, low expression of *let-7e* may be a biomarker of non-small cell lung cancer [28]. Also, *let-7e* expression is significantly reduced in retinoblastoma [29], and low *let-7e* expression contributes to cisplatin resistance in epithelial ovarian cancer [30]. To explore the function of diminished *let-7e* expression in GSCs, we studied the effects of restoring or inhibiting of *let-7e* expression in GSCs. Our results indicated that overexpression of *let-7e* suppressed the proliferation, migration and invasion and promoted the apoptosis of GSCs, whereas inhibition of *let-7e* facilitated the malignant progression of GSCs. In addition, *NRAS* expression was reduced in GSCs that overexpressed *let-7e*.

LncRNAs have been confirmed to function as endogenous miRNA sponges by binding to miRNAs and restraining their function [31]. We used bioinformatics software (Starbase) to determine whether *NEAT1* harbors a *let-7e* binding site, and identified two putative binding sites (Figure 5B). Additional experiments revealed that *let-7e* expression was significantly rescued when *NEAT1* was inhibited. *Let-7e* overexpression reduced the expression of *NEAT1,* while *let-7e* inhibition increased *NEAT1* expression. This suggested that *NEAT1* and *let-7e* participate in a reciprocal repression feedback loop. Dual-luciferase reporter assays verified that *let-7e* binds to *NEAT1* in a sequence-specific manner. Further, an RIP experiment confirmed the involvement of the RISC complex in the reciprocal repression between *NEAT1* and *let-7e*. This finding was consistent with a previous report that *XIST* functions as tumor suppressor by upregulating *miR-152* in a RISC-dependent manner, and harbors a *miR-152* binding site that allows *XIST* function as a ceRNA of *miR-152* in GBM stem cells [32]. *HOTAIR* is confirmed to promote the malignancy of renal carcinoma cells, and to stimulate oncogenesis by suppressing *miR-141* in the RISC complex [33].

To validate our hypothesis that *NEAT1* promoted the malignant progression of GSCs by suppressing *let-7e*, we established GSCs in which *NEAT1* was stably inhibited and *let-7e* was overexpressed or inhibited. The inhibition of *let-7e* in these cells largely reversed the suppression of GSC progression resulting from the knockdown of *NEAT1*. Furthermore, in *NEAT1*-knockdown GSCs, the inhibition of *let-7e* restored *NRAS* expression. These results indicated that *NEAT1* promotes oncogenesis by downregulating *let-7e* in GSCs.

MiRNAs regulate cellular events by binding to the 3′-UTRs of downstream genes. For instance, *miR-186* inhibits GSC growth by binding to the 3′-UTRs of *XIAP* and *PAK7*[34]. As our bioinformatics analysis and dual-luciferase assay demonstrated, *NRAS* is a direct target of *let-7e* and promotes malignant behavior in GSCs. For over four decades, scientists have searched for a safe and effective method of hindering the aberrant activity of RAS in human cancers [22]. *NRAS* belongs to the *RAS* gene family, and its encoded protein is essential for controlling the activity of several cellular signaling pathways, including PI3K/AKT/mTOR and MEK/ERK [35–37]. It is well known that the PI3K/AKT/mTOR and MEK/ERK pathways can directly and effectively promote cancer cell proliferation, migration and invasion, while inhibiting the apoptosis of tumor cells [38–41]. NRAS has been confirmed to activate these two important pathways, suggesting that therapies targeting *NRAS* may be promising anticancer treatment methods. Moreover, high-frequency *NRAS* mutations occur in various tumors, such as melanoma, juvenile myelomonocytic leukemia and colorectal cancer [42, 43]. *NRAS* mutations contribute to the response to cetuximab treatment in colorectal cancer cells by reducing patients survival [43]. Localized immunotherapies exhibit enhanced activity in high-grade melanoma and may be especially effective in those with *NRAS* mutations [44]. Further, NRAS protein levels are dysregulated in several tumor cells. *NRAS* is overexpressed in lung cancer cells and can be directly downregulated by *miR-515-5p* [45]. *MiR-421* can inhibit prostate cancer progression by attenuating NRAS protein expression [46]. Dual-luciferase reporter assays confirmed our hypothesis that *let-7e* could bind to *NRAS* in a sequence-specific manner, and *NRAS* expression was inversely affected by the overexpression or downregulation of *let-7e* in GSCs. Importantly, the *NRAS* 3′-UTR reversed the *let-7e*-induced impairment of malignant behavior in GSCs. Whether NRAS protein is a viable clinical therapy target for patients with glioma remains to be investigated.

In conclusion, we have shown that the knockdown of *NEAT1* impaired malignant behavior in GSCs by upregulating *let-7e* expression. Moreover, *let-7e* suppressed GSC malignant behavior by binding to *NRAS*. The significance of the correlation among *NEAT1*,*let-7e* and *NRAS* expression was highlighted for the first time. Thus, therapies targeting the *NEAT1*/*let-7e*/*NRAS* axis may be promising options for the treatment of human glioma.

## MATERIALS AND METHODS

### Clinical tissues

All glioma tissues and normal brain tissues (NBTs) were collected from surgical resections of human glioma in the Department of Neurosurgery, Shengjing Hospital of China Medical of University, from January 2013 to September 2015. Informed consent was gathered from all patients, and the research method was approved by the Ethics Committee of Shengjing Hospital of China Medical University. Grades of glioma were assigned by neuropathologists according to World Health Organization (WHO) classification. These tissues were divided into four groups: grade I (n=30), grade II (n=30), grade III (n=30) and grade IV (n=30). NBTs were used as the negative control group (n=30), and were obtained from patients who had been in traffic accidents that required immediate partial resections of brain tissue to reduce intracranial pressure, but unfortunately died after surgery. The tissues we obtained were far from the trauma tissue. All informed consent forms were obtained from relatives of the trauma victims. The specimens were immediately frozen and preserved in liquid nitrogen until use in this research.

### Cell culture

Human glioma cell lines (U87, T98G, U251, A272 and U373) and human embryonic kidney (HEK) 293T cells were purchased from the Chinese Academy of Medical Sciences (Beijing, China). They were cultured in high-glucose Dulbecco's Modified Eagle Medium (DMEM) with 10% fetal bovine serum (FBS, Gibco, Carlsbad, CA, USA). Primary normal human astrocytes were purchased from Sciencell Research Laboratories (Carlsbad, CA, USA) and cultured in astrocyte medium (Sciencell Research Laboratories). All cells were incubated at 37°C in a humidified incubator with 5% CO_2_.

### Isolation and identification of GSCs

GSCs were obtained and isolated as described previously [47]. Briefly, GSCs were cultured in DMEM/F-12 medium (Life Technologies Corporation, Grand Island, NY, USA) supplemented with basic fibroblast growth factor (20 ng/mL, Life Technologies Corporation, Carlsbad, CA, USA), epidermal growth factor (20 ng/mL, Life Technologies Corporation, Gaithersburg, MD, USA) and 2% B27 (Life Technologies Corporation, Grand Island, NY, USA). As previously described, sphere cells were dissociated in 96-well plates for the limiting dilution assay and primary sphere formation assay [48, 49]. Sphere cells were plated onto glass coverslips coated with poly-L-ornithine (BD Biosciences, Franklin Lakes, NJ, USA) in medium containing 10% FBS for the differentiation assay. For immunostaining of undifferentiated spheres, cells were incubated with antibodies against Nestin and CD133 (also known as prominin-1) (1:100, Santa Cruz Biotechnology, Santa Cruz, CA, USA). For immunostaining of differentiated spheres, cells were stained with antibodies against GFAP (1:100, Abcam, Cambridge, MA, USA) and beta-tubulin III (1:100, Santa Cruz Biotechnology). The primary antibody complexes were visualized with anti-rabbit Alexa Fluor 488 and anti-mouse Alexa Fluor 555 secondary antibodies (Beyotime Institute of Biotechnology, Jiangsu, China). Nuclei were counterstained with 4′, 6-diamidino-2-phenylindole (DAPI).

### Tumor xenografts

Four-week-old male nude mice were purchased from the National Laboratory Animal Center (Beijing, China). All mice were given free access to autoclaved food and water during the experiment. All experiments with nude mice were performed strictly in accordance with a protocol approved by the Administrative Panel on Laboratory Animal Care of the China Medical University. The mice were subcutaneously injected with 5 × 10^4^ GSC-U87 (or GSC-U251) and non-GSC-U87 (or non-GSC-U251) cells (n = 6 in each group). The subcutaneous tumor-bearing mice were sacrificed six weeks after injection. The tumor volume was calculated by the formula: volume (mm^3^) = length × width^2^/2.

### Cell transfections

The *NEAT1* knockdown (sh-*NEAT1*) plasmid and the respective non-targeting sequence (negative control, sh-NC), as well as the *let-7e* agomir (pre-*let-7e*), *let-7e* antagomir (anti-*let-7e*) and their respective non-targeting sequences (negative controls: pre-NC and anti-NC) were synthesized (GenePharma, Shanghai, China). The *NRAS* full-length (with 3′-UTR) plasmid (*NRAS* (+)), short-hairpin *NRAS* plasmid (*NRAS* (−)), *NRAS* (without 3′-UTR) plasmid (*NRAS* (non-3′UTR)) and their respective non-targeting sequences (negative controls: *NRAS* (+)-NC and *NRAS* (−)-NC) were synthesized (Life technology, Carlsbad, CA, USA). Cells were transfected through the use of Opti-MEM and Lipofectamine 3000 (Life Technologies Corporation, Carlsbad, CA, USA) according to the manufacturer's instructions when cells were at 50-70% confluence. The applicable stably transfected cell lines were established by selection with G418 screening. The transfection efficiency was verified by qRT-PCR.

### RNA extraction and qRT-PCR

Total RNA was extracted from clinical specimens and cells with the Trizol reagent (Life Technologies Corporation, Carlsbad, CA, USA). The primers for *NEAT1* and *GAPDH* were synthesized by Takara Bio (Japan). A One-Step SYBR PrimeScript RT-PCR Kit (Perfect Real Time) (Takara Bio) was used for qRT-PCR. The primers for *NEAT1*: forward 5′-ATGCCACAACGCAGATTGAT-3′, reverse 5′-CGAGAAACGCACAAGAAGG-3′; *GAPDH*: forward 5′-TGCACCACCAACTGCTTAGC-3′, reverse 5′-GGCATGCACTGTGGTCATGAG-3′. cDNA was generated from miRNA with a TaqMan miRNA Reverse Transcription kit (Applied Biosystems, Foster City, CA, USA). TaqMan Universal Master Mix II was used to perform TaqMan microRNA assays for *let-7e* and *U6* (Applied Biosystems, Foster City, CA, USA) on the ABI 7500 Fast Real-Time PCR System (Applied Biosystems). *GAPDH* and *U6* were used as endogenous controls for gene and miRNA expression, respectively. Gene and miRNA expression were normalized to those of the respective endogenous controls, and the fold-change in gene expression was calculated as 2^−ΔΔCt^.

### Cell proliferation assay

After transfection efficacy was confirmed, GSCs were dissociated with Accutase (Life Technologies Corporation, Carlsbad, CA, USA), resuspended, and seeded in 96-well plates at 3000 cells per well. The CCK-8 assay (Dojin, Japan) was used to measure GSC proliferation. CCK-8 solution (10 μL) was added to each well and the plate was incubated for 2 h at 37°C. The absorbance was recorded at 450 nm on a SpectraMax M5 microplate reader (Molecular Devices, USA).

### Cell migration and invasion assay

A 24-well insert with an 8-mm pore size (Corning, USA) was employed for the GSC migration and invasion assays. GSCs were dissociated with Accutase, resuspended in 100 μL serum-free medium and placed in the upper chamber (without or pre-coated with 500 ng/mL Matrigel solution (BD, Franklin Lakes, NJ, USA) for the migration and invasion assays, respectively), while 600 μL of 10% FBS medium was placed in the lower chamber. After incubation at 37°C for 48 h, the cells on the upper membrane surface were scraped off. The cells on the lower side of the member were fixed and then stained with 10% Giemsa. Cells were counted from five random vision fields under a microscope for statistics.

### 3D Spheroid-based tumor migration assays

For further analysis of the invasion abilities of GSCs, 3D Spheroid-based tumor migration assays were performed. Briefly, a Matrigel matrix (BD, State of New Jersey) was added to each well of a 96-well black, clear-bottom spheroid microplate (Corning, New York). The plate was then transferred to a 37°C/5% CO_2_ incubator for 1 h to initiate gel formation. GSCs were diluted to a concentration of 3×10^4^/mL in medium, and 100 μL of this dilution was plated to each of the appropriate wells. After 48 h, cells were monitored from five random vision fields under a microscope for statistics.

### Apoptosis detection

Apoptosis was measured with Annexin V-PE/7AAD staining (Southern Biotech, Birmingham, AL, USA) according to the manufacturer's instructions. After being washed twice with 37°C PBS and stained with Annexin V-PE/7AAD, cells were analyzed by flow cytometry (FACScan, BD Biosciences), and apoptotic fractions were recorded with CELL Quest 3.0 software.

### Dual-luciferase reporter assays

*NEAT1* and *NRAS* 3′-UTR sequences were amplified by PCR and cloned into a pmirGlo Dual-luciferase miRNA Target Expression Vector (Promega, Madison, WI, USA) to construct luciferase reporter vectors (*NEAT1*-Wt and *NRAS*-Wt) (GenePharma). The theoretical *let-7e* binding sequences in *NEAT1* and *NRAS* were mutated as indicated (*NEAT1*-Mut and *NRAS*-Mut). HEK-293T cells were co-transfected with the combinations of plasmids described below when they were at 50-70% confluence. A dual-luciferase reporter assay kit (Promega) was used to determine the luciferase activity 48 h after transfection. For the *NEAT1* binding assay, the cells were divided into five groups: the control group, *NEAT1*-Wt+*let-7e*-NC group, *NEAT1*-Wt+*let-7e* group, *NEAT1*-Mut+*let-7e*-NC group, and *NEAT1*-Mut+*let-7e* group. Likewise, for the *NRAS* binding assay, the cells were divided into five groups: the control group, *NRAS*-Wt+*let-7e*-NC group, *NRAS*-Wt+*let-7e* group, *NRAS*-Mut+*let-7e*-NC group, and *NRAS*-Mut+*let-7e* group.

### RNA immunoprecipitation

To investigate whether *NEAT1* was associated with the RISC, we performed RNA immunoprecipitation. GSCs were lysed in complete RNA lysis buffer containing protease inhibitor and RNase inhibitor from an EZ-Magna RIP RNA-binding protein immunoprecipitation kit (Millipore, Billerica, MA, USA) according to the manufacturer's protocol. Whole cell lysates from the control groups and *let-7e* groups were incubated with RIP immunoprecipitation buffer containing magnetic beads conjugated with human anti-Argonaute 2 antibody (Millipore) and the negative control (normal mouse IgG; Millipore). Samples were incubated with Proteinase K buffer, and then the immunoprecipitated RNA was isolated. The RNA concentration was measured with a NanoDrop (Thermo Scientific) and the RNA quality was assessed with a bioanalyser (Agilent, Santa Clara, CA, USA). Purified RNA was obtained, and qRT-PCR was performed with the primers mentioned above to demonstrate the presence of the binding targets.

### Immunohistochemistry assays

Slides of specimens (4 μm thick) were dewaxed, rehydrated, and incubated in 0.3% H_2_O_2_ for 10 minutes to inhibit endogenous peroxidase activity. Slides were then blocked with 10% normal goat serum (MXB, Fuzhou, China) for 30 minutes and incubated overnight at 4°C with a rabbit polyclonal antibody against NRAS (1:50, Abcam, UK) and XIAP (1:50, Abcam, UK). Slides were washed with PBS three times and then incubated with biotinylated rabbit anti-rabbit IgG for 1 h at room temperature. After incubation with an avidin-biotin-peroxidase complex for 10 minutes, samples were stained with 3,3′-diaminobenzidine. Slides were imaged under a light microscope (Olympus, Japan) at 100× and 200× magnification.

### Western blot

Total proteins were extracted from cells with RIPA buffer containing protease inhibitors (Beyotime Institute of Biotechnology) on ice, and these proteins were then subjected to SDS-PAGE and electrophoretically transferred to PVDF membranes. After non-specific binding was blocked with 5% nonfat milk at room temperature for 2 h, membranes were incubated with primary antibodies as follows: NRAS (1:2000, Abcam, UK) and GAPDH (1:1000, Santa Cruz Biotechnology). Then, the membranes were incubated with HRP-conjugated secondary antibodies (1:5000 goat anti-rabbit or goat anti-mouse, respectively; Santa Cruz Biotechnology) at room temperature for 2 h. Immunoblots were visualized by enhanced chemiluminescence (ECL kit, Santa Cruz Biotechnology) and recorded with ChemImager 5500 V2.03 software. The relative integrated density values were calculated with GAPDH as an internal control.

### Statistical analysis

Data are presented as the mean ± standard deviation (SD). All experimental results were statistically analyzed with Student's t-test or one-way analysis of variance (ANOVA). All statistical analyses were performed with SPSS 18.0 statistical software, with *P*<0.05 considered as statistically significant.

## SUPPLEMENTARY FIGURE


