# Supplementary material for: Knockdown of NEAT1 restrained the malignant progression of glioma stem cells by activating microRNA let-7e
Source: Oncotarget. 2016 Aug 19;7(38):62208–23. doi: 10.18632/oncotarget.11403 (PMC5308721; doi:10.18632/oncotarget.11403)
Supplement: Supplementary file 1 [file oncotarget-07-62208-s001.pdf]

Knockdown of *NEAT1* restrained the malignant progression of glioma stem cells by activating microRNA *let-7e*

SUPPLEMENTARY FIGURE

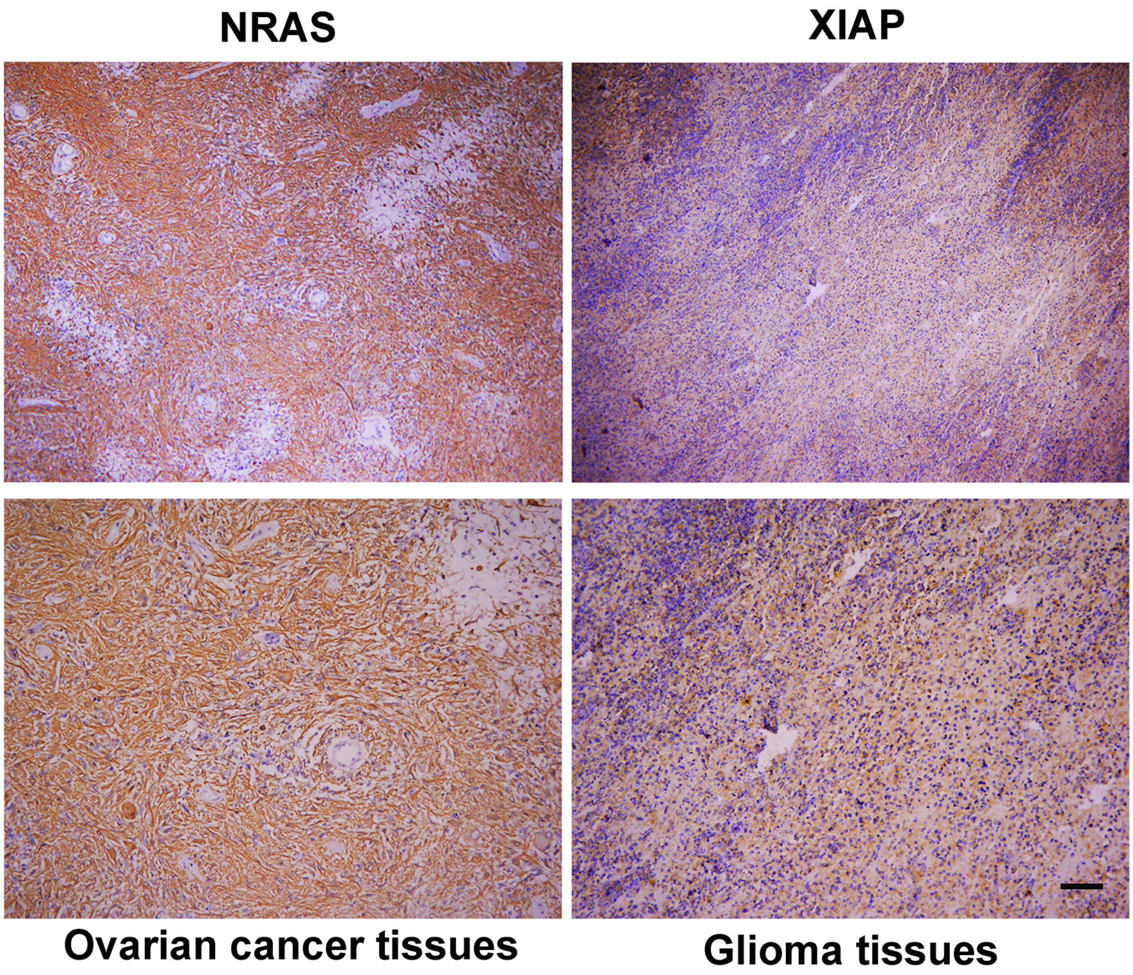

Supplementary Figure S1: Positive control tissue and a control primary antibody for Figure 5A. Left: Immunohistochemistry of *NRAS* protein in ovarian cancer tissue. Right: Immunohistochemistry of XIAP protein in glioma tissue. Scale bar, 50 μm.
